# Famous Poisoners in Fiction

**Published:** 1893-12-30

**Authors:** 


### Famous Poisoners in Fiction.

### VIII.—POISONER AND PHYSICIAN.

Very different from the rank of most of the novelists with which we have previously dealt is that of the writer from whose book we are about to take our poisoner for this article. Harrison Ainsworth could never by any possibility be called a genius; he is so plodding, so matter of fact, that he scarcely deserves the name of romancer, or its present day equivalent—novelist. Yet his works have a certain fascination just as a very plain spoken man gains sometimes attention by his sharp directness and uncouthness of speech and Harrison Ainsworth has many a votary, although they are usually of immature years. The "Star Chamber" is, perhaps, his most practical work. As usual, fiction and fact are strangely intermingled, and also, as usual, the facts are calmly perverted at the demand of fiction. That a poisoner such as Luke Hatton may well have lived in that age when poisoners were ever ready with their most dangerous weapon, and when the discoveries of science were not so far advanced as in these days, and detection, therefore, was easily defied, is indeed most probable; and that the physician's power was in the middle ages oftimes made to work for death and not for life, is the firm opinion of most historians. In the art of healing the instruments used are mostly preparations of dangerous poisons, and the medico holds in his hand a weapon more dangerous than the soldier's sabre or the executioner's axe. Why, then, must it be considered wondrous, remembering the temptations of the all-powerful God Mammon, and the cruel greed for gold which lies deep down in the heart of man, that the fictitious poisoner, Luke Hatton, had most likely more than one prototype in once living men? Indeed, it is believed by most English historians that a Luke Hatton, poisoner and physician, really did ply his awful trade during the 17th century. It was for money he killed; for money he saved life.

The following, Ainsworth's description of his poisoner, is, like his books, passable at first sight, but lacking the depth and sublimity of genius. "His face was like an ugly mask, on which a sardonic grin was stamped. His features were large and gaunt, and he had the long hooked nose, and the sharp-pointed bestial ears of a satyr, with leering eyes betokening at once sensuality and cunning. He had the skin and beard of a goat, and crisply-curled hair of a pale yellow colour. With all this there was something sordid in his looks as well as his attire, which showed that to his other vices he added that of avarice. A mock humility belied by the changeless sneer on his countenance distinguished his deportment. It could be seen at once that, however cringing he might be, he despised the person he addressed. Moreover, in spite of all his efforts to control it, there was something sarcastic in his speech. His doublet and hose, both of which had seen some service and were nearly thread-bare, were tawny-coloured, and he wore a short yellow cloak, a great ruff of the same colour, and carried a horn steeple-crowned hat in his hand." Such is the novelist's description of this 17th century satyr, this imp in human form, and except for the fairness of colouring so particularly Saxon, it would stand for a description of the famous Venetian poisoner Ruggiens, who, standing before the Council of Ten, used to calmly appraise his victims, according to their social position and the advantages of their death to the State.

Luke Hatton pleaded his claim as a poisoner well. "My draughts," he says, "have removed many a troublesome husband, and silenced many a jealous wife. I have helped many an heir to the speedy enjoyment of an inheritance which but for my assistance would not have come to him for years. The lover, with a rival in his way, who has come to me, has soon been freed from all anxiety on that score. The courtier, eager for a post which a superior held, has gained it by my aid." Surely our novelists speak truly when they call poison "a great power," and "a mighty, if silent, influence."

Many poisons have the same effect on the system as a fever, inducing inextinguishable thirst, burning pains, &c., and there is nothing in Ainsworth's account of the poisoning of Lady Roos to indicate any special poison. The cordial which proves a counter-poison is one also which is unknown to the medical profession, and is most likely only the product of a fertile brain. Its effects are rather too magical even for an antidote, although indeed in this weird world truth is often stranger than fiction, and in the region of medical science there have been discovered wondrous facts, which a man sceptically inclined would declare to be utter nonsense, impossible, and perfectly untrue. And yet the medical Professors of to-day are inclined to doubt, because the clue to them is lost, the existence of many a poison of the fifteenth and sixteenth century.

Feared, obeyed, courted, this poisoner of the 17th century most likely died a rich man, richer perchance than any of our nineteenth century physicians who use poisons only for the relief of suffering and the cure of the diseased and dying. Not so many years after the events related in the book, King Charles himself, dying rather suddenly, was supposed by many to have been poisoned, the accused being his brother, the hated Duke of York. There is little doubt than this rumour, combined with the fact of his being of a persecuting frame of mind, and a rigid Roman Catholic, cost him his throne and drove him into exile. There does not, however, appear to have been any reasonable grounds for the supposition; but suspicion and fear were rife in the land, and every one dreaded his neighbour.

The whole horror of the fear of poison lies in its mysteriousness. It strikes in the dark, and men, when fearful of it, see a foe in every shadow, an avenger in every turn of the path of life. It is also a fact for congratulation that secret poisoning, as a means of secret murder, is fast sinking into desuetude. The secret poisoner, to be successful, must be a person of education, or at least of intellectual capacity. But it is persons of this class who so keenly appreciate the risks of scientific detection. So long as practical science continues to advance, so long will secret poisoning be a dangerous and therefore a rare occupation.

